# The Pathway Tools Pathway Prediction Algorithm

**DOI:** 10.4056/sigs.1794338

**Published:** 2011-12-23

**Authors:** Peter D. Karp, Mario Latendresse, Ron Caspi

**Affiliations:** Bioinformatics Research Group, SRI International 333 Ravenswood Ave, EK207, Menlo Park, CA 94025

## Abstract

The PathoLogic component of the Pathway Tools software performs prediction of metabolic pathways in sequenced and annotated genomes. This article provides a detailed presentation of the PathoLogic algorithm. The algorithm consists of two phases. The reactome inference phase infers the reactions catalyzed by the organism from the set of enzymes present in the annotated genome. The pathway inference phase infers the metabolic pathways present in the organism from the reactions catalyzed by the organism. Both phases draw on the MetaCyc database of metabolic reactions and pathways. MetaCyc contains two data fields to support pathway inference: the expected taxonomic range of each pathway, and a list of key reactions for pathways. These fields have significantly increased the predictive accuracy of PathoLogic.

## Overview

We describe the metabolic pathway prediction algorithm used by the Pathway Tools software [1,2]. Pathway Tools is in use by a large number of groups to reconstruct the metabolic pathway complement of organisms from their annotated genomes, and to create curated metabolic pathway databases. For example, the SRI BioCyc database collection [3,4] contains Pathway/Genome Databases (PGDBs) for 1,004 genomes, and MicroCyc [5] contains 535 genomes. Curated Pathway Tools-based PGDBs are available for *Mus musculus* [6], *Saccharomyces cerevisiae* [7], *Arabidopsis thaliana* [8], *Drosophila melanogaster* [9], *Escherichia coli* [10], and *Homo sapiens* [11]. See [12] for a comprehensive listing of Web-accessible PGDBs.

The Pathway Tools pathway prediction algorithm predicts pathways in a sequenced genome by recognizing in that genome previously known pathways from the MetaCyc database [4]. Pathways are recognized based on the enzymes present in the genome. Pathway Tools does not perform sequence analysis and it does not predict the functions of the proteins in a genome. It assumes that the genome has already been annotated using one of the many available genome annotation pipelines, such as [13,14].

Pathway Tools contains several computational inference components [1], which are collectively called PathoLogic. Henceforth, this article will use the term PathoLogic to refer to the Pathway Tools pathway prediction algorithm.

## Background: The MetaCyc Pathway Database

**Figure 1:**
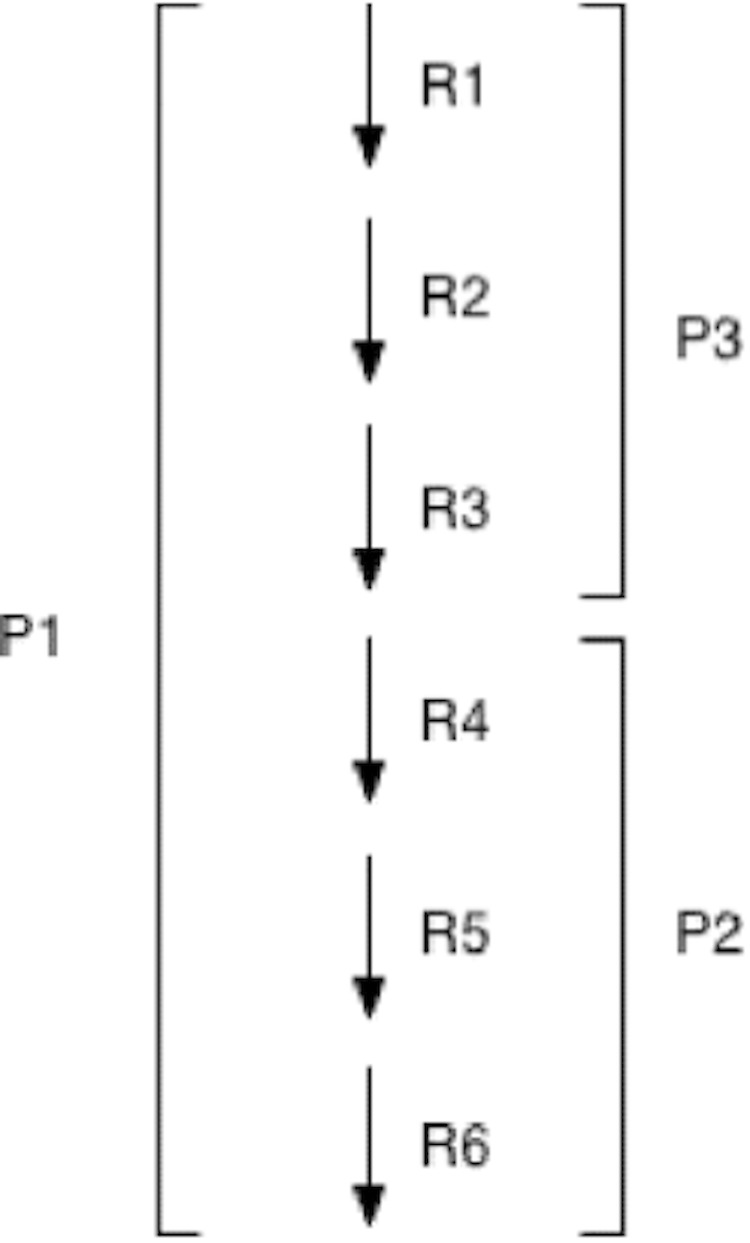
Example pathway illustrating how MetaCyc curators sometimes split pathways into smaller evolutionarily conserved units.

The MetaCyc metabolic database is the reference pathway database used by PathoLogic for pathway prediction [Figure 1]. Version 14.6 (released in November 2010) of MetaCyc contains 1,583 base metabolic pathways and 263 superpathways.

The pathway information in MetaCyc has been specially honed to support pathway prediction in the following respects. First, curators sometimes divide pathways into smaller segments that correspond to atomic units conserved by evolution. This process is informed by curators observations of which pathway segments occur in different organisms, and can yield more accurate pathway predictions. For example, imagine that organism A contains a linear pathway P1 consisting of six consecutive reactions, R1–R6. Imagine, however, that organism B contains a smaller portion of P1 consisting of reactions R4–R6, which we will call P2. If P1 were present in MetaCyc and we were performing a pathway prediction in organism B, and B contained enzymes for reactions R4–R6, we would predict P1 as present in B. However, this inference would be incorrect because B in fact contains the shorter pathway P2. When MetaCyc curators encounter this situation, they define pathway P2 in MetaCyc, and a new pathway P3 consisting of reactions R1–R3. Pathway P1 will be redefined as a superpathway containing P2 and P3. In this way PathoLogic can properly recognize whichever of P1, P2, or P3 is present in a given genome.

A second way in which MetaCyc is optimized for pathway prediction is that it contains information on the expected taxonomic groups in which most MetaCyc pathways occur. Curators have recorded this taxonomic range information (in slot Taxonomic–Range) at whatever granularity they deem appropriate to capture their expectation of the taxonomic groups in which the pathway will be found. Taxonomic range assignments vary from very broad (e.g., “Bacteria”) to very specific (e.g., “Mycoplasma”), and are based on both the taxonomic groups in which the pathway is known to occur, and the function of the pathway. The next section describes how PathoLogic uses taxonomic range information.

MetaCyc curators sometimes specify key reactions within a pathway. Key reactions are used by PathoLogic to differentiate between related pathway variants. Key reactions are reactions whose presence is considered as a critical signature for the presence of the pathway.

MetaCyc contains a large collection of enzyme names obtained from the Enzyme Nomenclature List prepared by the International Union of Biochemistry and Molecular Biology [15] and from the MetaCyc curators. Approximately 24,000 enzyme names are present in MetaCyc version 14.6.

## Procedures Used

Pathway prediction is divided into two phases: prediction of the reactome (reaction complement) of the organism, and prediction of pathway complement of the organism from its predicted reactome.

### Input to PathoLogic

The input to PathoLogic is an annotated genome. The genome is provided as a set of files. For each replicon or contig in the genome, the user provides:

- An annotation file, in either Genbank format [16] or in PathoLogic format [17]
- A sequence file, formatted in FASTA format (Optional)

We have found that considerable variability exists in how different groups interpret the Genbank file format, and that it is not unusual to find Genbank files that are incompatible with the interpretation used by PathoLogic. Therefore, if Genbank format files are provided, care should be exercised in determining that proper tag names are used within the file (e.g., gene product names should be provided within the /product tag), and that information is formatted properly within each tag (e.g., product names such as “tryptophan synthetase” should not be suffixed or prefixed with additional information such as gene names, protein length, etc.). Exact details of expected file formats are provided in the Pathway Tools User’s Guide [17].

### Reactome Inference

The first step in pathway prediction is to infer the set of biochemical reactions that can be catalyzed by the enzymes encoded by the genome, that is, to infer the reactome of the organism. Because reactions are the direct building blocks of pathways, once we know the reactome of the organism, we can more easily assess the evidence for what pathways are present.

The output of the reactome inference phase is the set of inferred reactions. All inferred reactions and their substrates are copied from MetaCyc to the organism-specific PGDB.

The PathoLogic reactome inference module examines each gene product in the genome, and attempts to infer what if any reactions are catalyzed by that gene product. Often no reactions are inferred since many genes have no assigned function, and many genes that have assigned functions are not metabolic enzymes (e.g., transcription factors and histones do not have enzymatic activities).

The reactions catalyzed by a gene product are inferred from three information fields present in the Genbank or PathoLogic file for a gene product: the EC number, gene product name (enzyme name), and Gene Ontology (GO) terms. Ideally, the user would provide the gene product name plus either the EC number or GO terms (note that EC number and GO terms are redundant to a significant degree since a large number of Gene Ontology molecular function terms correspond directly to EC numbers). We prefer to have EC number or GO terms provided because these terms are from a controlled vocabulary and are therefore much easier to recognize than are uncontrolled enzyme names. However, many metabolic enzymes have not yet been assigned EC numbers or GO terms; therefore, controlled vocabulary terms cannot be assigned to many metabolic enzymes. For multifunctional enzymes, a gene product name and either EC number or GO terms should be provided for each enzyme function.

We emphasize that it is the preceding information only that is used to infer the reaction(s) catalyzed by each gene product. Sequence information is not used by PathoLogic in reactome inference. Note also that many reactions predicted by PathoLogic do not end up being attached to metabolic pathways after PathoLogic pathway inference, which can occur because a reaction is not a component of any pathway in MetaCyc, and because no pathway of which the reaction is a component was inferred as present in the PGDB.

The reactome inference algorithm infers the reactions catalyzed by protein P as follows.

1. For each EC number ECi attached to P, look up all reactions R annotated with ECi in MetaCyc. Infer that P catalyzes every reaction in R for each ECi.
2. If GO terms are attached to P, their associated EC numbers (if any) are also used in the previous step.
3. For each enzyme name (gene product name) ENi attached to P, perform a lookup of ENi in MetaCyc. If a single matching reaction is associated with ENi, infer that P catalyzes the reaction associated with ENi in MetaCyc. The lookup of ENi in MetaCyc involves the following processing:
  - Convert ENi to lower case and remove punctuation characters such as space and hyphen to eliminate variation in styling of enzyme names.
  - Look up ENi in MetaCyc. If found, declare a match and stop any further searching. If not found, try the following step.
  - Remove various prefixes and suffixes, one at a time, from ENi that can interfere with matching such as “putative,” “hypothetical,” “predicted,” “precursor,” “secreted,” and many other qualifiers, some based on regular expressions. For each removal, search for the reduced name in MetaCyc. Once a match is found, declare a match and stop further searching.
  - If the same enzyme name matches multiple reactions, flag the name as ambiguous and do not infer reactions for it.

The reactome inference module also compares the reactions inferred from enzyme name, EC numbers, and GO terms to ensure that they are consistent. If, for example, the enzyme name and EC number identify reaction sets with a null intersection, then a warning is produced, and PathoLogic infers the reaction for the EC number.

### Pathway Inference

Pathway inference is based on the set of catalyzed reactions that were imported from MetaCyc during the reactome inference step. All pathways inferred during the phase described have also come from a fixed reference database, namely MetaCyc. In the following when we say a pathway is inferred, it means that PathoLogic has predicted that the pathway is present in this organism. All inferred pathways are copied from MetaCyc to the PGDB being generated for this organism. When we say a reaction is present in the generated PGDB, we mean the reaction has been inferred as present in the organism by the reactome inference step, or that the reaction is marked as spontaneous in MetaCyc.

Inference of a pathway is based on the approach that if no rule can be found to reject (i.e., not infer) a pathway, then the pathway should be inferred as present in that PGDB. PathoLogic applies the following rules to infer pathways. The rules are applied to base pathways (not to super pathways) individually and in no particular order.

**Rules for inferring base pathways:**
1. If a pathway is mostly absent in the generated PGDB, it is not inferred. A pathway is mostly absent if it has more than one missing reaction in the generated PGDB, and if it has no more than one reaction present.
2. A reaction is unique (to a pathway) if the reaction is used in only one pathway in the generated PGDB. If a pathway P has a unique reaction, considering the pathways that were not inferred, P is inferred. In the particular case that two or more pathways share a unique reaction among them (meaning the reaction is found only in that set of pathways), and the only reason to keep these pathways is based on the uniqueness of that reaction, then one of the pathways is randomly selected to be kept.
3. A biosynthetic pathway that is a proper subset of some other pathway and is missing its final two reactions is not inferred. Similarly, a degradative pathway that is a proper subset of some other pathway and is missing its initial step is not inferred. The intuition behind the preceding rules is that multiple biosynthetic pathways sometimes share common initial steps, and it is the final steps that are most indicative of the presence of the pathway (and conversely for catabolic pathways). An energy metabolism pathway with no unique reactions having fewer than half of the reactions present in the generated PGDB is not inferred.
4. If the reactions in a pathway P1 are a superset of the reactions of an already inferred pathway P2, and P1 has more missing reactions than P2, P1 is not inferred.
5. MetaCyc pathways contain taxonomic range assignments that indicate the range of organisms in which a pathway is likely to be found. Because MetaCyc taxonomic range assignments are approximate and are based on incomplete knowledge, PathoLogic does allow pathways to be inferred in organisms outside their taxonomic range, but it requires very stringent evidence to do so. A pathway outside its taxonomic range will be predicted as present if and only if all reactions in the pathway are present in the generated PGDB. The use of the taxonomic range information can be deactivated by the user.
6. Many pathways of MetaCyc have some reactions designated as key reactions. Any pathway that is missing at least one key reaction in the generated PGDB is not inferred. If all key reactions of a pathway are present in the generated PGDB, the pathway is inferred.
7. Electron transfer pathways are inferred if and only if they are complete, that is if and only if all their reactions are present in the generated PGDB. No other electron transport pathways are inferred.

### Rules for inferring superpathways:

1. MetaCyc contains superpathways composed of individual (base) pathways, or a combination of base pathways and superpathways. A MetaCyc superpathway is inferred in the generated PGDB if and only if all its component superpathways and base pathways are present in the generated PGDB. Since superpathways can be part of other super-pathways, the decision to include or exclude a superpathway is performed recursively starting from the superpathways that do not include any child superpathways.

## Discussion

An evaluation of the PathoLogic algorithm performed one year ago [2] using a gold-standard pathway database found its accuracy ((true positives plus true negatives) divided by all examples) to be 91%, its specificity (true negatives divided by all negatives) to be 94%, its precision (true positives divided by (true positives plus false positives)) to be 77.9%, and its sensitivity (true positives divided by all positives) to be 79.3%. Since then we have made a number of small improvements to the algorithm that have likely improved its accuracy [1].

As MetaCyc has grown in size in the last several years it has been a challenge to maintain the accuracy of PathoLogic. The introduction of key reactions and taxonomic range information in MetaCyc increased the accuracy of PathoLogic substantially. We have recently improved the speed of PathoLogic by approximately a factor of 5 to decrease the time it requires to process large metagenomics datasets. To our knowledge, the pathway prediction algorithms used by KEGG and Reactome have not been published. Therefore, we cannot compare our algorithm to theirs.

PathoLogic can generate a “pathway evidence report” Web page that lists all pathways it has predicted in an organism and the evidence supporting each predicted pathway (example page [18] ). This report provides a convenient way for a scientist to review the evidence for each pathway. The pathways are grouped by the MetaCyc pathway ontology. For each predicted pathway the report shows a low-resolution pathway diagram that indicates which reactions in the pathway are present in the organism, the total count of reactions present, the number of reactions that are used in other pathways, and a list of those other pathways.

## Availability

MetaCyc is freely and openly available to all users. Pathway Tools runs on Linux, Windows, and Macintosh; it is freely available to academic users, and is available to commercial users for a fee. MetaCyc and Pathway Tools may be obtained from [19].

## References

[1] Karp PD Paley SM Krummenacker M Latendresse M Dale JM Lee T Kaipa P Gilham F Spaulding A Popescu L Pathway Tools version 13.0: Integrated software for pathway/genome informatics and systems biology. Brief Bioinform 2010; 11:40-79; . 10.1093/bib/bbp043 19955237 PMC2810111

[2] Dale JM Popescu L Karp PD Machine learning methods for metabolic pathway prediction. BMC Bioinformatics 2010; 11:15 10.1186/1471-2105-11-15 20064214 PMC3146072

[3] BioCyc Database Collection http://BioCyc.org/

[4] Caspi R Altman T Dale JM Dreher K Fulcher CA Gilham F Kaipa P Karthikeyan AS Kothari A Krummenacker M The MetaCyc database of metabolic pathways and enzymes and the BioCyc collection of pathway/genome databases. Nucleic Acids Res 2010; 38:D473-D479 10.1093/nar/gkp875 19850718 PMC2808959

[5] MicroCyc home page. https://www.genoscope.cns.fr/agc/microscope/metabolism/microcyc.php

[6] MouseCyc Database http://mousecyc.jax.org:8000/

[7] YeastCyc Database http://pathway.yeastgenome.org/

[8] AraCyc Database http://www.arabidopsis.org/biocyc/

[9] FlyCyc Database http://biocyc.org/FLY/organism-summary?object=FLY

[10] EcoCyc Database http://EcoCyc.org/

[11] HumanCyc Database http://HumanCyc.org/

[12] Pathway/Genome Database Websites http://BioCyc.org/otherpgdbs.shtml

[13] IGS Annotation Engine home page. http://ae.igs.umaryland.edu/cgi/index.cgi

[14] Stewart AC Osborne B Read TD DIYA: a bacterial annotation pipeline for any genomics lab. Bioinformatics 2009; 25:962-963 10.1093/bioinformatics/btp097 19254921 PMC2660880

[15] ExplorEnz home page. http://www.enzyme-database.org/

[16] Genbank Format http://www.ncbi.nlm.nih.gov/collab/FT/#7.1.2

[17] Karp PD, Paley S. Pathway Tools User’s Guide version 7.0. Available from SRI International, 2003.

[18] Pathway Evidence Report for Bacillus subtilis. http://biocyc.org/BSUB/hierarchical.html

[19] BioCyc Downloads http://biocyc.org/download.shtml

